# Cost of referral treatment for colic in the United Kingdom—What has changed in the last 5 years?

**DOI:** 10.1111/evj.70074

**Published:** 2025-08-18

**Authors:** F. E. Wilson, T. S. Mair, S. L. Freeman

**Affiliations:** ^1^ School of Veterinary Medicine and Science University of Nottingham Leicestershire UK; ^2^ Bell Equine Veterinary Clinic Kent UK

**Keywords:** euthanasia, horse, insurance, medical, readability, surgery

## Abstract

**Background:**

Referral treatment costs and insurance status impact treatment decisions for colic.

**Objectives:**

To evaluate changes in the cost of referral treatment for colic, and insurance cover and premiums in the United Kingdom between 2018 and 2023.

**Study Design:**

Cross sectional study.

**Methods:**

Thirty UK equine referral hospitals were contacted in January 2024 and asked about their colic caseload and costs of the last three cases across six categories (surgical +/− resection, euthanasia before, during or after surgery, and medical treatment), using similar methodology to a 2018 study. Data are reported as mean/median (range). A standardised case was used to retrieve data on veterinary fees, insurance cover, and monthly premiums from five companies. Findings were compared with actual and inflation‐adjusted 2018 data. Readability of insurance documents were assessed using the Flesch Kincaid Reading Ease (FKRE) score and the Gunning Fog Score (GFS). The FKRE is ranked from 0 to 100 (easy to read‐hard to read); FKRE scores below are 65 recommended. The GFS estimates the years of formal education needed to understand text; GFS scores higher than 12 are too complex for most people to read.

**Results:**

Eighteen hospitals responded, contributing costings for 248 cases in total. Mean/median (range) costs for cases euthanised without surgery (*n* = 41) were £1200 (£500–£4389), for medical cases (*n* = 44) were £2379 (£683–£13,762), and for all surgical cases that survived surgery (*n* = 122) were £7905 (£3023–£20,343). When compared with inflation‐adjusted 2018 data, medical treatment and euthanasia without surgery costs had increased; surgery costs had decreased. Maximum insurance cover was between £5000 and £7500. The actual cover value had not changed for 3/5 companies since 2018, and was reduced for 4/5 companies after inflation adjustment. Monthly premiums ranged from £42.76 to £97.23, and were all increased compared with 2018 inflation‐adjusted data (£34.01–£59.39). Insurance document FKRE Scores ranged from 31.2 to 54.8, and GFS ranged from 13.6 to 20.6. All were outside the recommended range.

**Main Limitations:**

Small case numbers, UK population only.

**Conclusions:**

Costs of referral treatment have largely risen in line with inflation, and now frequently exceed maximum insurance cover. Insurance premiums have increased above inflation, and insurance documents remain complex and hard to read.

## 1 INTRODUCTION

Approximately one in five cases of colic will be critical, requiring referral treatment for intensive medical therapy, or surgery, or euthanasia.^1^ The costs associated with this can be high and can be a major factor affecting horse owners' decisions.^2^ A previous study documented a decline in the number of horses undergoing colic surgery between 2004 and 2017, with rising costs thought to be an underlying cause.^3^ In 2018, Barker documented costs for referral colic treatment across a sample of UK hospitals and identified that these often exceeded current insurance policy limits.^4^ Insurance documentation can be difficult for owners to understand, with legal terminology and complex sentence structures affecting readability.^5^ There are a number of tools which can be used to measure readability, including the Flesch Kincaid and Gunning Fog Scores.^6^, ^7^ The Flesch Kincaid Reading Ease is the most widely used measure of readability. The score is ranked from 0 to 100, and the higher the score, the easier the text is to read. For most businesses, a score of 65 is recommended.^8^ The Gunning Fog Score estimates the years of formal education needed to comprehend text. A good score is suggested as 7/8; scores higher than 12 are too complex for most people to read, and scores higher than 17 are graduate level texts.^9^ The study by Barker in 2018 used the Flesch Kincaid and Gunning Fog Scores to analyse the readability of key insurance documentation (including the policy details and the terms and conditions) from five companies.^4^ The findings were that most documents were highly complex and difficult to read and understand. The aims of this study were to evaluate any changes to the cost of referral treatment for colic and any changes in the cost of insurance cover and the readability of key insurance documents over the last 5 years.

The objectives were:

1. To document the current (2023–2024) costs of referral treatment for colic (including medical treatment, surgical treatment and euthanasia) in UK equine hospitals, and compare these to the costs of referral treatment obtained in 2018.
2. To evaluate the cost of insurance cover in the United Kingdom for a standardised case example in 2024 and compare this with costs for a similar case obtained in 2018.
3. To evaluate the readability scores of current (2024) insurance cover and premiums for five insurance companies, and compare these to the readability scores obtained for the same insurance companies 5 years previously (2018).

## 2 METHODS

### 2.1 Objective 1—Costs of referral treatment for colic

The study used similar methodology to the previous study in 2018,^4^ but with additional data collected around each hospital's case numbers, surgeons and approaches to fees. The surgical cases were subcategorised into additional divisions compared with the 2018 data to enable data to be collected on cases with and without intestinal resection.

Study population and sampling: An online survey was distributed to a lead contact at each hospital listed as an accredited equine hospital in the United Kingdom by the Royal College of Veterinary Surgeons on 23 January 2024,^10^ with follow‐up reminders at 2–4 week intervals until 29 February 2024.

Variables: Survey participants were asked to complete anonymised data on their referral caseload for horses with colic, including whether participants thought there had been any changes in caseload and selection of treatment options over the previous 5 years, whether their hospitals had a fixed fee for colics, and whether their hospitals discounted any fees for colics. They were also asked to provide anonymised data on the total bill for the last three cases seen within the following categories:

- Colic surgery without intestinal resection; horse discharged home.
- Colic surgery with intestinal resection, horse discharged home.
- Colic surgery with intestinal surgery; horse died before discharge.
- Colic cases euthanised without surgery.
- Colic surgery, horse euthanised on the table.
- Colic not treated surgically.

The insurance status of the horses was not recorded.

Data analysis: Normality of data was assessed by visual assessment of frequency histograms. Descriptive analysis was performed (mean/median and range) on all data categories. Range is reported rather than standard deviation or standard error of mean for all data, as it was felt that the minimum and maximum values for each data set would be most valuable for horse owners/carers and veterinary surgeons. If data sets were incomplete (e.g., not completed for some categories), this was reported as (*n*). The number and frequency percentage of cases are reported to the nearest whole number, and the cost data are reported to the nearest GB pound. The 2023/24 data were compared with data previously obtained in 2018 where applicable.^4^ The actual costs in 2018 and the inflation adjusted costs have been reported. The inflation adjusted costs are the costs from 2018 with the inflation added for their comparable cost in 2024, using the Bank of England inflation calculator.^11^

### 2.2 Objectives 2 and 3—Insurance cover and policies

The study repeated the methodology of the previous study in 2018.^4^ A standardised case example was used to request quotes for insurance cover in January 2024, using the same horse details (14‐year‐old 15.1hh Welsh Section D gelding) and the same insurance companies (NFU Mutual, The Insurance Emporium (previously E and L), KBIS, SEIB and PetPlan) as previously.^4^ Data on the amount covered for veterinary fees, loss of use and disposal, ages insured, method and ease of obtaining a quote, the monthly premium quoted, and the excess payable were obtained and compared with the 2018 data. The ease of obtaining a quote was evaluated using a scale one to five (one = 1–10 questions, two = 11–20 questions, three = 21–30 questions, four = 31–40 questions, five = 40 or more questions). The readability of the insurance terms and conditions and product information documents were assessed by the WebFX readability assessment tool using the Flesch Kincaid Reading Ease Score and Gunning Fog Score (www.webfx.com/tools/read-able/). The 2023/24 data were compared with the data previously obtained in 2018.^4^ The actual costs in 2018 and the inflation adjusted costs have been reported. The inflation adjusted costs are the costs from 2018 with the inflation added for their comparable cost in 2024, using the Bank of England inflation calculator.^11^

## 3 RESULTS

### 3.1 Objective 1—Costs of referral treatment for colic

Twenty‐nine of the 30 hospitals contacted were eligible to participate; one closed during the study. Eighteen hospitals returned data, including costs for a total of 248 cases referred for colic. The number of colic surgeries performed annually was a mean of 41 (range 10–100). Based on participants' opinions of trends, the average number of colic surgeries performed over the previous 5 years had stayed the same for 7/17 hospitals, gone down for six hospitals, and gone up for 4 hospitals. Discounts were given on colic treatment by 13/17 hospitals (76.5%), including three hospitals which had a fixed fee option. Fixed fee options included fixed fees for admission assessments and for specified surgical and post‐operative treatments (e.g., 5‐ or 7‐days hospitalisation). Reasons for discounts were not specified.

**FIGURE 1:**
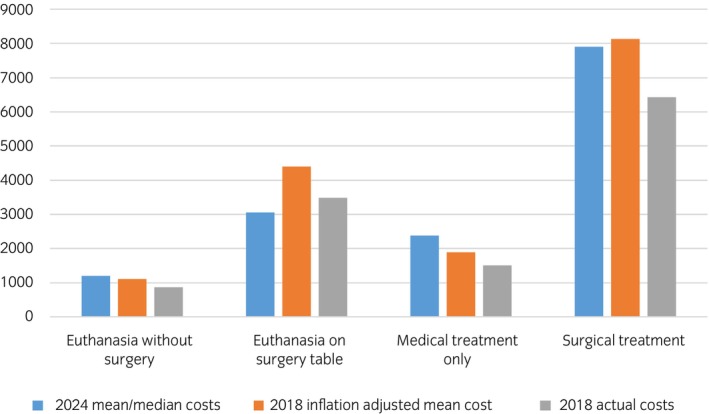
Mean/median cost of referral colic treatment at 18 UK equine hospitals in 2023/4 (*n* = 248 horses total), compared with data from 2018 (*n* = 108). (Data for 2018 are presented as actual costs, and inflation adjusted costs to account for inflation rates between 2018 and 2024).

The mean/median cost of treatments for horses undergoing referral colic treatment, with comparative data from 2018, is reported in Table 1 and Figure 1. The costs for all referral options had increased from 2018 to 2023/24 for all categories except for cases that were euthanised during or after surgery (Table 1, Figure 1). The median cost of horses that were euthanised within the first 24 h without surgery had increased slightly from a mean inflation‐adjusted cost of £1104 (mean actual cost of £874) in 2018 to a median £1200 in 2023/24. The mean cost of medical treatment had increased from a mean inflation‐adjusted cost of £1897 (mean actual cost of £1501) to £2379 in 2023/24 (Table 1, Figure 1).

**TABLE 1:** Mean/median cost of referral colic treatment and range of costs at 18 UK equine hospitals for six different outcomes of an episode of colic 2023/4 (*n* = 248 horses total).

Colic outcome	Mean (range) cost (2023/4)^a^	Mean cost (2018)—Actual cost	Mean cost (2018) adjusted for inflation rates between 2018 and 2024^b^
Outcome 1: euthanasia (*n* = 41)	£1200^a^ (£500–£4389)	£874 (£460–£1472)	£1104 (£581–£1860)
Outcome 2: surgery and euthanasia (*n* = 41)	£3063^a^ (£1500–£6740)	£3485 (£1580–£10,301)	£4404 (£1997–£13,017)
Outcome 3: medical treatment (*n* = 44)	£2379 (£683–£13,762)	£1501 (£554–£3821)	£1897 (£700–£4829)
Outcome 4: surgical treatment no resection (*n* = 45)	£7049 (£4094–£20,112)	n/a	
Outcome 5: surgical treatment with resection (*n* = 41)	£8025 (£4813–£12,001)	n/a	
Outcome 6: surgical treatment, died (*n* = 36)	£8841 (£3,023–£20,343)	n/a	
Outcome 7: all surgical treatment	*£7905* (£3023–£20,343)	£6437 (£3179–£9100)	£8134 (£3179–£11,500)

*Note:* Individual numbers for each outcome are reported in the table. Data are compared with actual data and inflation‐adjusted costs 2018 data (*n* = 108 horses). Outcome 1: cases euthanised within the first 24 h of hospital admission without any surgical intervention. Outcome 2: cases euthanised during/after first surgical intervention. Outcome 3: cases that received medical intervention and survived more than 24 h after hospital admission. Outcome 4: cases that received surgical intervention without resection and survived to discharge (2023/4 data only). Outcome 5: cases that received surgical intervention with resection and survived to discharge (2023/4 data only). Outcome 6: cases that received surgical intervention and did not survive to discharge (2023/4 data only). Outcome 7: cases that received surgical intervention and survived more than 24 h after hospital admission, combining 2023/4 data from outcomes 4, 5, 6 with comparable 2018 data. n/a: not available.

^a^ Non‐normal data are reported as median.

^b^ Inflation adjusted to calculate cost of 2018 data in 2024 was calculated using the Bank of England inflation calculator.^11^

The costs for cases that received colic surgery in 2023/24 (a) with intestinal resection and survived to discharge was £8025, (b) without intestinal resection and survived to discharge was £7049, and (c) with intestinal resection and died/euthanised post‐operatively before discharge was £8841 (Table 1). There were a total of 122 horses across these surgery categories (including those with and without resection, and those that were euthanised before discharge or survived to discharge). The mean cost for all these surgery cases was £7905. This was compared with the 2018 data which only reported surgery (without the sub categories relating to resection or survival to discharge), which was a mean inflation‐adjusted cost of £8134 (mean actual cost of £6437) (Table 1, Figure 1). There were 111/122 (91.0%) of the surgical cases in 2023/24 (including those with and without resection, and those that survived to discharge or were euthanised/died after surgery and before discharge) which had bills in excess of £5000. Using the same surgical case grouping, there were 62/122 (50.8%) cases that had bills in excess of £7500. The mean cost for cases which were euthanised during the first surgery had decreased from a mean inflation‐adjusted cost of £4404 (mean actual cost of £3485) in 2018 to £3125 in 2023/24 (Table 1, Figure 1).

### 3.2 Objective 2—Cost of insurance cover

Maximum veterinary fees cover ranged from £5000 to £ 7500for the five companies that provided quotes (Table 2). The veterinary fees cover provided by the insurance companies was unchanged for three companies and increased for two companies since 2018 when actual costs were compared. One company had increased cover from £5750 to £ 7000, and another had a similar cover of £5000 but had added a maximum cover of £7500 for colic surgery since 2018 (Table 2). When inflation‐adjusted costs for 2018 were compared with 2024 data, then all veterinary fees cover had decreased apart from the company which had added additional cover for colic surgery. The cover available for loss of use varied between companies from 60% to 100% of the value of the horse and was largely similar to 2018. The amount of cover available for body disposal cost ranged from £175 to £300 (Table 2). Cover available for body disposal cost was unchanged from the actual costs for 2018 for three companies, increased with one company, and decreased for one company. When 2018 inflation‐adjusted costs for body disposal were compared with 2024 costs, four of the five companies cover had decreased. The maximum age of horse which could be insured ranged from 19 to 30 years, with one company stating there was no limit; this was largely unchanged since 2018 (Table 2).

**TABLE 2:** The highest level of veterinary fees cover offered, cover offered for loss of use and payment for disposal for five UK insurance companies, using a standardised equine case submitted in 2024.

Insurance company	Maximum veterinary fees cover offered (2018 actual cost data; inflation adjusted cost^a^)	Cover available for loss of use of horse	Amount of cover available for cost of body disposal	Ages of horse which can be insured
NFU mutual	£5000 (2018 data: £5000 actual costs; £6319 inflation adjusted)	–^b^ (2018 data: 80%)	£250 (2018 data: £250 actual costs; £316 inflation adjusted)	30 days to 19 years (2018 data: 30 days to 19 years policy inception, covered till 25 years)
The Insurance Emporium (was E&L)	£7000^c^ (2018 data: £5750 actual costs; £8846 inflation adjusted)	60% or 100%^d^ (2018 data: 100%)	£300 (2018 data: £200 actual costs; £253 inflation adjusted)	31 days to 20 years (2018 data: 31 days to 15 years)
KBIS	£7500 (2018 data: £7500 actual costs; £9478 inflation adjusted)	–^e^ (2018 data: not available)	£200 (2018 data: £200 actual costs; £253 inflation adjusted)	24 h to 30 years (2018 data: 90 days to 20 years)
SEIB	£5000 (£7500 for colic surgery)^f^ (2018 data: £5000 actual costs; £6319 inflation adjusted)	75% (2018 data: 60 or 75%)	£175 (2018 data: £300 actual costs; £379 inflation adjusted)	30 days to no limit (2018 data: 30 days to not specified on policy)
PetPlan	£5000 (2018 data: £5000 actual costs; £6319 inflation adjusted)	60% (2018 data: 60 or 100%)	£200 (2018 data: £200 actual costs; £253 inflation adjusted)	Up to 25 years (2018 data: 30 days to 19 years policy inception, covered till 25 years)

*Note*: The 2024 data are compared with actual data and inflation‐adjusted data obtained in 2018 using the same standardised case. KBIS—will cover £7500 for colic surgery on a catastrophe cover policy, but non‐surgical treatment of colic is not included. Cover for surgical and non‐surgical colic treatment is £3000. (Further details: Catastrophe cover is separate to leisure horse cover. Catastrophe can be purchased on its own for up to £7500 for colic surgery. Type B leisure horse cover is up to £7500 on colic surgery, but for surgery costs only. Type C leisure horse cover is up to 75% of vet fees OR 100% for colic surgery (after excess removed) up to £3000 per incident).

^a^ Inflation adjusted to calculate cost of 2018 data in 2024 was calculated using the Bank of England inflation calculator.^11^

^b^ Loss of use only available up to 12 years old.

^c^ £1000 for complementary treatment and £500 for transport to referral vets is applied to all cover levels.

^d^ Complete loss of use is sum insured/market value (whichever is less). Partial loss of use is 60% sum insured/market value (whichever is less).

^e^ No permanent loss of use cover available. Only available for competition insurance.

^f^ Veterinary fees are insured up to £5000 under standard policy. Catastrophe policy has cover up to £7500 for colic surgery, but non‐surgical treatment of colic is not included.

The monthly premium that would be paid by an owner for insurance cover for an identical horse case example to that used in 2018 had increased for all five insurance companies (Table 3). The range of monthly premium costs in 2024 was £42.76–£97.23 compared with 2018 inflation‐adjusted monthly premiums ranging from £34.01 to £59.39 (actual data from 2018 ranged from £27.06 to £47.06). All had increased compared with the inflation‐adjusted 2018 monthly premiums; premiums for two companies were more than double the inflation‐adjusted 2018 figures (Table 3). The ease of obtaining a quote was unchanged for three companies, increased for one company, and decreased for one company; two companies required over 40 questions answering in 2024 (one company only in 2018) (Table 3).

**TABLE 3:** Method and ease of obtaining a quote, cost for monthly premium payable, and the excess payable for five UK equine insurance companies, using a standardised case example submitted in 2024.

Insurance company	Method of obtaining quote	Ease of obtaining quote^a^	2024 quote for example horse premium per month	2018 quote for example horse premium per month (inflation adjusted costs^b^)	2024 excess payable by owner	2018 excess payable by owner (inflation adjusted costs)
NFU mutual	Telephone	5	£97.23	£47.06 (£59.39)	£200	£145 (£183)
The insurance emporium	Online	3	£74.51	£27.06 (£34.01)	£375	£159 (£201)
KBIS	Online	3	£42.76	£30.02 (£37.91)	£140	£140 (£177)
SEIB	Online	3	£95.83	£33.19 (£41.70)	£200^c^	£165 (£209)
PetPlan^d^	Online	3	£66.95	£35.44 (£44.23)	£145	£145 (£183)

*Note*: The 2024 data are compared with actual data and inflation‐adjusted data obtained in 2018 using the same standardised case.

^a^ Scale for ease of obtaining a quote was: 1 = 1–10 questions, 2 = 11–20 questions, 3 = 21–30 questions, 4 = 31–40 questions, 5 = >40 questions.

^b^ Inflation adjusted to calculate cost of 2018 data in 2024 was calculated using the Bank of England inflation calculator.^11^ The calculator does not allow values less than £1.00 to be entered so the monthly premiums for 2018 were rounded to nearest pound for calculation.

^c^ SEIB offer a range of excesses from £200 to £750. £200 was used as most similar to the other policies.

^d^ Petplan offers a free month of cover when you buy online instead of over the phone.

### 3.3 Objective 3—Readability of insurance documentation

The terms and conditions for each policy were assessed using the Flesch Kincaid Reading Ease score and the Gunning Fog score (Table 4). The Flesch Kincaid Reading Ease scores varied from 31.2 to 54.8 in 2024 (range 21.6–57.7 in 2018). No terms and conditions documents met the recommended score for businesses. The Gunning Fog score varied from 13.7 to 19.8 in 2024 (range 12.4–20.8 in 2018). All companies had a Gunning Fog score above 12, and 3/5 had a score higher than 17. Four companies had over 15% of the text as complex words, and for one company, 20% of the words were complex (Table 4).

**TABLE 4:** Readability of the terms and conditions documents provided by UK equine insurance companies assessed in January 2025 using the WebFX readability assessment tool.

Name of company	Flesch Kincaid Reading ease score^a^	Gunning fog score^a^	Number of words	Percentage of complex words	Age easily understood by (years)
NFU Mutual	40.1	17	15,780	16.12%	19–20
The Insurance Emporium	36.2	19.8	8590	18.47%	21–22
KBIS	38.6	17.7	15,362	18.62%	19–20
SEIB	30.8	17.2	13,512	20.54%	21–22
PetPlan	54.8	13.7	15,592	14.13%	16–17

^a^ The Flesch Kincaid Reading Ease is ranked from 0 to 100 (hard to read‐easy to read). For most businesses, a score of 65 is recommended. The Gunning Fog Score estimates the years of formal education needed to comprehend text. A good score is suggested as 7/8; scores higher than 12 are too complex for most people to read; scores higher than 17 are graduate level texts.

The policy information documents for each company were also assessed using the Flesch Kincaid Reading ease score and the Gunning Fog score (Table 4). Two companies (PetPlan and SEIB) did not have a product information document; the details of the cover page on their website were used instead. The Flesch Kincaid Reading Ease scores varied from 28.8 to 47.9 in 2024 (range 28.7–64.9 in 2018). The Gunning Fog Score varied from 13.6 to 20.6 in 2024 (range 6.6–20.7 in 2018). All five companies had over 15% of the text as complex words, and for two companies, 20% of the words were complex (Table 5).

**TABLE 5:** Readability of the product information documents provided by UK insurance companies assessed in January 2025 using the WebFX readability assessment tool.

Name of company	Flesch Kincaid Reading ease score^a^	Gunning fog score^a^	Number of words	Percentage of complex words	Age easily understood by (years)
NFU Mutual	44.3	15.1	1060	18.11%	18–19
The Insurance Emporium	33.5	19.5	1207	18.39%	21–22
KBIS	43.7	15.8	1613	20.09%	18–19
SEIB	47.9	13.6	490	16.94%	17–18
PetPlan	28.8	20.6	269	20.45%	22–23

^a^ The Flesch Kincaid Reading Ease is ranked from 0 to 100 (hard to read‐easy to read). For most businesses, a score of 65 is recommended. The Gunning Fog Score estimates the years of formal education needed to comprehend text. A good score is suggested as 7/8; scores higher than 12 are too complex for most people to read; scores higher than 17 are graduate level texts.

PetPlan had the hardest‐to‐read policy document, but the easiest‐to‐read terms and conditions, similar to the findings in 2018. SEIB was the easiest‐to‐read policy document, but one of the hardest‐to‐read terms and conditions.

## 4 DISCUSSION

This study gives a snapshot of current costs for colic surgery and equine insurance options in the United Kingdom in 2023/2024. When compared with the 2018 study, these data also show the changes that have occurred over the previous 5‐year period and how this relates to UK inflation rates for goods and services.^4^ When adjusted according to UK inflation rates over this period, the cost of medical treatment had increased compared with inflation rates. There was little change in the euthanised only costs, but the relative costs of horses undergoing surgery and for horses euthanised at surgery had decreased. The level of insurance maximum cover was largely unchanged from 2018, and therefore when adjusted for inflation rates, the maximum cover was less than that available in 2018 for 4/5 companies. The costs of insurance premiums had increased above the rate of inflation for all companies over the past 5 years, with premiums for two companies over double the costs from 2018, even when adjusted for inflation rates. Over 90% of all the surgical cases had referral costs over £5000, and for many cases, the insurance cover will not be sufficient to cover the cost of referral surgery. The gap between insurance cover and treatment cost appears to have further increased for animals insured with some policies since 2018.^4^ The readability of the insurance policy documents and terms and conditions remained very difficult to read. There was minimal change from 2018, and it is likely that a large number of owners will not fully understand this documentation. The current situation is therefore that the cost of surgical referral treatment increased in line with inflation; the insurance cover available for most companies is lower relative to 2018 inflation‐adjusted figures. The cost of insurance premiums has concurrently risen above inflation rates, and insurance documents remain difficult to read and understand.

There are numerous factors which have changed for equine veterinary practices in the United Kingdom since 2018, including increasing corporatisation,^12^ increasing employment costs, changes to working hours, Brexit,^13^ shortages of equine veterinary surgeons,^14^, ^15^ cost of living crisis, and rising costs of amenities and running costs.^16^ The observed increase in the actual costs of colic treatment agree with the findings of Elane et al. (2024) who identified increasing costs for horses undergoing surgical treatment for colic at two equine hospitals (one in the United Kingdom and one in the United States) between 2013 and 2023.^17^ The factors contributing to the cost of colic treatment, include the running costs of the veterinary practices, the salaries of the veterinary and support team, as well as the cost of drugs, consumables, diagnostic tests and treatments. New evidence and advances in diagnostic tests and treatment options are also likely to increase costs, but there are also major costs associated with maintaining or upgrading some of the supportive services around colic cases (e.g., anaesthesia equipment, and ultrasound machines). The rates of inflation in the United Kingdom have varied between 0.85% and 9.07% since 2018,^18^ and percentage change in salaries across the United Kingdom has been between 3.9% and 4.1% per year.^19^ Inflation for veterinary services was reported by which to have outstripped overall inflation over the last 2 years.^20^ Their report stated that cost of small animal annual booster injections had ‘increased more steeply over the past 2 years compared to private health services for humans’.^20^ They focused on these data, and described it as being the only data available from the Office of National Statistics. They reported a steady rise in the cost of vaccinations between 2010 and 2020 with a more rapid increase since the Covid‐19 pandemic, ‘equating to a percentage increase of 48.4% since January 2020 (by comparison the consumer price index increased by 20.4% during the same period)’.^20^ These changes are challenging for both veterinary practices and clients. In the UK press, there has been much discussion about the rise in veterinary fees. A news article in 2024 reported over 50% rises in cost of veterinary treatment, again based on data from the UK Office of National Statistics.^21^ A 2025 article reported 60% rises in vets' bills between 2015 and 2023 based on figures from the UK Competition and Markets Authority.^22^ Both these rates are much higher than the rates of inflation during this period (average inflation rate of 3.70% per year giving a cumulative price increase of 33.72% using the CPI Inflation Calculator UK Inflation Calculator: GBP from 1751 to 2025). The current study evaluated inflation rates over a shorter period of time (2018–2024), but there was a higher average inflation rate of 4.14% per year during this period, giving a cumulative price increase of 27.59%. In the current study, the inflation‐adjusted comparison of 2023/24 colic referral treatments costs with 2018 figures showed that there was minimal or no increase above inflation rates, apart from medical treatment. The costs for euthanasia during colic surgery had decreased both in terms of actual costs and the inflation adjusted costs. These findings are going against the data and trends reported in the UK veterinary sector as a whole.

In this current 2023/2024 data, over 75% (13/17) of the participating hospitals stated that they gave some discounted fees for colic cases. The survey did not ask for further information on this, and therefore it is unknown what cases were being discounted and how these discounts were being applied. Further work will be needed to explore this. A possible explanation is that practices are aware of the cost challenges and are trying to help with this. In the 2018 study, whether practices discounted fees was not investigated; therefore, it is not known whether this has increased. The category of horses that were euthanised during surgery showed a decrease in both the actual cost and inflation‐adjusted cost since 2018, which was somewhat surprising. There are several possible explanations for this. These cases could be most likely to have discounts applied due to the loss to the owner and emotional impact on all involved, including the owner and veterinary team. Clients may also be making euthanasia decisions sooner, due to increasing predicted costs and the mismatch between costs and insurance cover. Some hospitals may also be subsidising surgical fees, and the overall increasing costs for colic treatment may relate to the medical care and post‐operative intensive treatments. It has been suggested that one reason why overall costs of colic treatment have risen may be related to clinics and clinicians adding postoperative treatments or monitoring that may be considered standard of care, but that may not necessarily affect treatment plans or prognosis in a ‘kitchen sink’ effect.^23^ These data in this study, however, suggest that colic referral treatment is not seeing the same levels of increase above inflation rates as other areas of the veterinary sector.

The cost of colic surgery is an issue affecting decisions for both owners and veterinary surgeons in the United Kingdom.^2^, ^24^ The overall impact of this across the UK horse population is unclear. The trends reported by participants in this study for the number of horses undergoing surgery varied across the hospitals. Some hospitals reported increases in surgery cases in the past 5 years, some reported decreases, and some reported no changes. A study by Blikslager and Mair (2020) reported on the trends in the management of colic for North Carolina State in the United States and Bell Equine in the United Kingdom over a 14‐year period (2004–2017).^3^ Both hospitals documented an increase in costs, an overall trend of decreasing numbers of horses undergoing surgical treatment, and an increase in euthanised cases across the 14 years.^3^ The varying trends in the current study may be affected by several factors, such as location, hospital, changes in local hospitals and referring practices, and the numbers of horses being registered or treated at each practice. Further research is required to investigate this.

If horses are insured for veterinary fees, then the impact of costs is less influential on treatment decisions. A qualitative study documented that if the horse was insured, then the owners were more likely to opt for surgical treatment, and the finances were less influential on the ultimate decision.^24^ It described, however, that some owners felt that having insurance meant they had less control over the decision‐making, including a description of feeling like being on a ‘conveyor belt’.^24^ The impact of the insurance status is likely to be becoming less important. In the current study, the actual maximum cover available to the owner had increased in two companies and remained the same for three, and when adjusted for inflation rates within this period, the cover was relatively lower for four of the five companies. The gap between the maximum insurance cover available and the average costs for surgery has therefore widened. This is despite a substantial increase in premiums across all insurance companies evaluated, even when inflation‐adjusted data were considered. Insurance covers a range of diseases, and the overall cost of premiums has to cover increasing costs for these. For many colic cases, however, existing insurance cover will not be sufficient to cover the cost of referral surgery. This means that having insurance cover reduces, but does not remove, the financial component of decision‐making for clients. The impact of insurance will depend on the policy and the cover available. Options such as life‐time insurance for individual conditions and proportional contributions from owners were not available in the United Kingdom at the time of the 2018 study but are now available.^25^ This study repeated the same methodology as was used in 2018, so analysed cover and policies from the same companies that were researched in 2018. A wider range of insurance options is now available within the United Kingdom, and different options are also available across other countries.^26^ The insurance data presented here are specific to a defined case study and specific companies, and again further exploration is needed.

The readability of insurance documentation was essentially unchanged since 2018, and remains hard for most of the UK population to read and understand. The Organisation for Economic Co‐operation and Development (OECD) survey published in 2024 reported that ‘1 in 6 adults in England (i.e. 6.6 million people) have very low literacy skills’.^27^ This was defined as at or below Level 1 (Level 1 is equivalent to GCSE grades D‐G. Adults with skills below Level 1 may not be able to read bus or train timetables or understand their pay slip).^9^ There are a number of changes that can be made to reading materials to improve readability—these include avoiding technical terms, avoiding terms with multiple syllables, and keeping sentences short and concise with single points.^28^ Implementing these simple changes to insurance documentation can help owners choose the best options for themselves, and clearly understand what is and is not covered with each policy.^5^ Accessibility of information for people with a range of different backgrounds and needs is also being increasingly recognised as important. There have been advances in our understanding of specific learning difficulties. The use of alternatives to text, such as infographics can be beneficial, and should also be considered in the future to improve inclusivity.^29^

Study limitations included the geographical location and number of cases. The data were collected from UK hospitals only, and further studies investigating these trends internationally would be valuable. Participating practices were only requested to contribute data from small numbers of cases over a short period of time, to encourage participation. The study was dependent on participating practices freely contributing their time and information, and we therefore aimed to minimise this burden. There was a high response rate and engagement with the study (18/29 hospitals (62%) contributed data), for which we are hugely grateful. A larger number of practices contributing more data over a longer period of time would add to the strength of evidence, but would require a large time contribution from participants. We also did not ask practices to provide additional information, including case details such as type of resection, presence or absence of post‐operative complications, whether the horse was insured, and whether finance was a limiting factor in case decisions. All these factors are likely to influence decision‐making and overall costs, and further studies investigating these would be beneficial. An additional limitation to the interpretation of the financial data relates to the fact that these data do not include costs associated with the initial assessment of colic cases seen prior to referral to a hospital, and in some cases, this may include multiple visits and treatments prior to referral.

The data analysed within this study may help with advising an owner of the average costs for a specific category of colic in the United Kingdom; however, there was a large range in all categories investigated, which makes it challenging to advise owners of possible maximum costs for an individual case. Overall, the costs for referral treatment of colic in the United Kingdom have risen in line with inflation over the past 5 years, with treatment for medical cases increasing above inflation, and costs for horses euthanised during surgery now below inflation levels. Costs for most of the surgery cases, and some of the medical cases exceeded the standard insurance veterinary fees cover limits for the five companies that were researched. This study highlights that horse owners may have to contribute a considerable amount of their own finances, even if their horse is insured with the current costs and cover levels. Most insurance cover for veterinary fees had not increased in line with inflation and therefore is lower relative to those available 5 years ago. Insurance premiums, however, had increased above inflation rates for all five companies, with two of the five companies having monthly premiums more than double the inflation adjusted rates for the same standardised case example submitted 5 years ago. The insurance documents reviewed were difficult to read, and there has been minimal change in this over the last 5 years. Many owners will find it difficult to understand this documentation, and a change is needed to make this more accessible to the horse‐owning population.

## FUNDING INFORMATION

Not applicable.

## CONFLICT OF INTEREST STATEMENT

Tim Mair is employed by CVS Ltd.

## AUTHOR CONTRIBUTIONS

**F. E. Wilson:** Investigation; writing – original draft; writing – review and editing; visualization; formal analysis. **T. S. Mair:** Conceptualization; investigation; methodology; writing – review and editing; writing – original draft; supervision; visualization. **S. L. Freeman:** Conceptualization; investigation; methodology; writing – original draft; writing – review and editing; supervision; validation; visualization; formal analysis; data curation.

## DATA INTEGRITY STATEMENT

Sarah Freeman had full access to the data and takes responsibility for the integrity of the data and the accuracy of the data analysis.

## ETHICAL ANIMAL RESEARCH

The study was reviewed and approved by the Committee for Animal Research and Ethics (CARE), School of Veterinary Medicine and Science, University of Nottingham (Ref: 4048 240116).

## INFORMED CONSENT

All participating hospitals gave informed consent for use of their data.

## Data Availability

The data that support the findings of this study are available upon reasonable request from the corresponding author. Open data sharing exemption granted by the editor.
